# Reconstruction of *Eriocheir sinensis* Y-organ Genome-Scale Metabolic Network and Differential Analysis After Eyestalk Ablation

**DOI:** 10.3389/fgene.2020.532492

**Published:** 2020-09-25

**Authors:** Bin Wang, Jiarui Yang, Chenchen Gao, Tong Hao, Jingjing Li, Jinsheng Sun

**Affiliations:** ^1^Tianjin Key Laboratory of Animal and Plant Resistance, College of Life Sciences, Tianjin Normal University, Tianjin, China; ^2^Tianjin Fisheries Research Institute, Tianjin, China

**Keywords:** *Eriocheir sinensis*, genome-scale metabolic network, ecdysterone synthesis, Y-organ, eyestalk ablation

## Abstract

Genome-scale metabolic network (GSMN) has been proven to be a useful tool for the system analysis of organism metabolism and applied to deeply explore the metabolic functions or mechanisms in many organisms, including model or non-model organisms. However, the systematic studies on the metabolisms of aquatic animals are seldom reported, especially the aquatic crustaceans. In this work, we reconstructed an *Eriocheir sinensis* Y-organ GSMN based on the transcriptome sequencing of Y-organ, which includes 1,645 reactions, 1,885 unigenes, and 1,524 metabolites distributed in 100 pathways and 11 subsystems. Functional module and centrality analysis of the GSMN show the main metabolic functions of Y-organ. Further analysis of the differentially expressed unigenes in Y-organ after eyestalk ablation reveals that 191 genes in the network were up-regulated and 283 were down-regulated. The unigenes associated with the ecdysone synthetic pathway were all up-regulated, which is consistent with the report on the increasing secretion of ecdysone after eyestalk ablation. Besides, we compared the Y-organ GSMN with that of *E. sinensis* eyestalk and hepatopancreas, and we analyzed the specific metabolisms in each organ. The specific metabolisms and pathways of these three networks are closely related to their corresponding metabolic functions. The GSMN reconstructed in this work provides a new method and many novel clues for further understanding the physiological function of Y-organ. It also supplies a new platform for the investigation of the interactions among different organs in the growth process of *E. sinensis*.

## Introduction

*Eriocheir sinensis* (Chinese mitten crab) is an important economic aquatic animal in China. The growth of *E. sinensis* undergoes multiple molting processes, which is regulated by the ecdysone and molt-inhibiting hormone. The main function of Y-organ is to secrete the precursor of ecdysone, which enters the blood and transforms to active ecdysone catalyzing by 20-hydroxyecdysone (Pu et al., 2015; Techa and Chung, 2015). The research of Y-organ and ecdysone mainly focuses on the gene clone and expression. Gong et al. (2014) cloned full-length cDNA sequence of a ecdysteroid receptor gene Ers-EcR from *E. sinensis* Y-organs and found that it has the highest expressions in Y-organ and muscle. He et al. (2013) constructed an expression vector of *E. sinensis* ecdysone regulation protein (ERP) using homologous reorganization and obtained the purified ERP. However, it is noticed that the metabolism or regulation in cells interacted in a complex biological system, in which the function of each gene is influenced by other genes or proteins. Therefore, a systematic view is necessary for the function analysis and related regulation studies in Y-organ.

As an efficient tool for the systematic investigation of the complex metabolic systems, genome-scale metabolic networks (GSMNs) were reconstructed for more than 100 organisms including archaea, bacteria, and lots of eukaryotes. GSMN has been applied to metabolic engineering, bioenergy development, and biomedicine. The standard protocol of the reconstruction of GSMN was reported by Thiele and Palsson (2010), which greatly promoted the development of network reconstruction. It provides a new method for quickly and efficiently searching for metabolic pathways and for prediction of gene functions or gene phenotypes. However, the GSMN reconstruction and analysis of the aquatic animals are seldom reported. In the previous research, the GSMNs of *E. sinensis* eyestalk (Wang B. et al., 2016) and hepatopancreas (Hao et al., 2018) were reconstructed. In recent years, researchers paid many efforts to analyze the metabolisms and regulation in *E. sinensis* based on homologous gene alignment, homology searches (Ding et al., 2018), transcriptome assembly, annotation, and differential expression analysis (Chen et al., 2017). However, in the growth process of *E. sinensis*, the exact metabolic mechanism of each organ and the interaction between them are not clear.

In this work, we reconstructed a GSMN of *E. sinensis* Y-organ and analyzed its metabolic features. The differentially expressed unigenes (DEUs) in Y-organ after eyestalk ablation were further analyzed based on the GSMN to explore the interaction between Y-organ and eyestalk. We investigated the common and unique metabolisms of the three organs, compared with the previously reconstructed GSMN of *E. sinensis* eyestalk and hepatopancreas. The GSMN reconstructed in this work provides a new method and novel clues for further understanding the physiological function and mechanism of Y-organ.

## Materials and Methods

### Preparation of High-Through Sequencing Data

#### RNA Sequencing of Y-organ

Healthy crabs (body weight 5–6 g) with good vitality were held in fresh water at 18–20°C for 3–5 days. Sixty crabs were randomly selected and equally divided into two groups. The eyestalk of experimental group (C1) was ablated, and the Y-organ was taken as a sample 48 h later. The eyestalk of control group (C0) was not treated. The sample was stored in −80°C liquid nitrogen until RNA was extracted. The total RNA from the Y-organ was extracted using the TRIzol method (Invitrogen, United States) according to the manufacturer’s protocol.

After extraction of the total RNA from the samples, mRNA was enriched by using the oligo magnetic beads. By adding the fragmentation buffer, the mRNA was interrupted to short fragments (about 200 bp), and then the first-strand cDNA was synthesized by random hexamer-primer using the mRNA fragments as templates. Buffer, dNTPs, RNase H, and DNA polymerase I were added to synthesize the second strand. The double-stranded cDNA was purified with QiaQuick PCR extraction kit and washed with elution buffer (EB) for end repair and single nucleotide A (adenine) addition. Finally, sequencing adaptors were ligated to the fragments. The required fragments were purified by agarose gel electrophoresis and enriched by PCR amplification. The cDNA libraries of two samples were sequenced using Illumina HiSeq^TM^2000 with single-end sequencing.

The original image data were transferred into sequence data by base calling, which is defined as raw reads. To obtain the clean reads, all raw reads were filtered to remove dirty reads, including reads with adaptors, reads in which unknown bases are more than 10%, and low-quality reads (the percentage of the low-quality bases of quality value ≤ 5 is more than 50% in a read). The clean reads obtained with sequencing were mapped to reference sequences obtained from transcriptome sequencing (ENA accession number: PRJEB4541) (Sun et al., 2014) using SOAPaligner/soap2 (Li et al., 2009) to get the unigenes. Unigenes with one or two base mismatches were collected in the alignment. The function annotation of unigenes was also extracted from the transcriptome sequencing including corresponding gene symbol, gene function, gene ontology (GO) and Kyoto Encyclopedia of Genes and Genomes (KEGG) orthology (KO) annotation. KO is a functional ortholog manually defined in the context of the KEGG molecular network (Kanehisa et al., 2016). A KO associates with different biological objects in the KEGG database related to the function, including genes, proteins, compounds, reactions, and pathways. Therefore, the association between different objects can be determined by the KO numbers.

The quality of the sequencing was further assessed by sequencing saturation analysis and distribution of reads on reference genes as well as clean read filter. Sequence saturation analysis is used to measure the sequencing data of a sample. When the number of reads increases, the number of detected genes also increases. However, when the number of reads reaches a certain value, the growth rate of detected genes flattens, and it means that the number of detected genes tends to saturation. Distribution of reads locating on the genes is used to evaluate the randomness. During the RNA-Seq experiment, mRNAs are firstly broken into short segments by chemical methods and then sequenced. If the randomness is poor, read preference from a specific gene region will directly affect subsequent bioinformatics analysis. Since reference genes have different lengths, the read location on gene is standardized to a relative position (which is calculated as the ratio between read location on the gene and gene length), and then the number of reads in each position is counted. If the randomness is good, the reads in every position would be evenly distributed.

The RNA-Seq data have been submitted to the Sequence Read Archive (SRA) database, and the accession number is SRX8639987 for sample C0^1^ and SRX8639988 for sample C1^2^. The unigene information of C0 sample obtained from differential gene expression (DGE) and transcriptome sequencing were used for the subsequent draft reconstruction of the GEM.

#### Differentially Expressed Unigenes in Y-organ

The unigene expression is calculated by the number of reads mapped to the reference sequence and every unigene. The unigene expression level was calculated by reads per kb per million (RPKM) (Basletic et al., 2008) and used in the DEU analysis. The statistical analysis of reads frequency was performed in C0 and C1 to screen the DEUs in *Eriocheir sinensis* Y-organ. Two criterions, false discovery rate (FDR) ≤ 0.001 and |log2 ratio(C1/C0)| > 1, were used to screen the DEUs. The differential expression data have been submitted to Gene Expression Omnibus (GEO) database, and the accession number is GSE153552^3^.

### Reconstruction of the *Eriocheir sinensis* Genome-Scale Metabolic Network

The reconstruction of the GSMN almost follows the standard protocol of GSMN proposed by Thiele and Palsson (2010). The reconstruction process is composed of draft reconstruction, refinement of reconstruction, conversion of reconstruction, network evaluation, and data assembly and dissemination.

#### Draft Reconstruction

The reaction list, metabolite list, reaction–KO relationships, and reaction_mapformula files were downloaded from the KEGG database. The unigene–KO relationship obtained from transcriptome data (ENA accession number: PRJEB4541) and information from the KEGG database were used as the data source or draft reconstruction. With KO as a bridge, the candidate metabolic reactions (including reaction identifiers, equations) and corresponding metabolites (including metabolite names and identifiers) from the KEGG database were matched with unigenes. With these reactions and metabolites, the draft reconstruction network was assembled.

#### Refinement of Reconstruction

##### Determination of chiral metabolites

In the draft reconstruction network, the same metabolite with different chirality was labeled with different compound IDs. For example, D-glucose with α and β chirality was labeled as C00267 and C00221, respectively. Besides, there is a D-glucose with unclear chirality labeled as C00031. These repetitive IDs for a metabolite may influence the accuracy of model simulation. Therefore, the chiral metabolites were revised. Taking glucose as an example, α-D-glucose is the predominant form of glucose in most organisms, so the glucose in the network was unified as alpha chirality.

##### Reference to the reactions in our previous model

As there is no *E. sinensis*-specific information in the KEGG database, we referenced to the reactions in our previous reconstructed eyestalk (Wang B. et al., 2016) and hepatopancreas GSMN of *E. sinensis* (Hao et al., 2018), in which the stoichiometry coefficient and directionality of some reactions have been revised. For the reactions that are common in Y-organ and the reference networks, the equations (including whole equation and main equation) and directions were directly taken from the reference networks.

##### Adding information for unigene and reaction localization

The compartment of metabolic reactions in our model was all considered to be cytoplasmic. Only the transport and exchange reactions were different. The compartments of substrates and products in the transport reactions were cytoplasm and extracellular, respectively, while the direction of the metabolic transport was determined by the positive and negative flux values of the transport reaction. The compartment of exchange reactions is extracellular.

##### Adding pathway and subsystem information

The pathway and subsystem information of reactions in the draft network were obtained from the KEGG database. For the reaction with multiple pathways, we selected the pathway in the main metabolic subsystem (usually the pathway with smallest number). A reaction has only one pathway in our network to facilitate the subsequent gap filling step.

##### Verify unigene–enzyme–reaction relationship

The enzyme–KO information was obtained from the KEGG database. In the daft reconstruction, the unigene–reaction relationship was determined by KO numbers. In this step, also taking KO number as a bridge, the unigene–enzyme–reaction relationships were identified. As unigene is not exactly the gene, the relationships between unigenes were difficult to be identified. In this work, all the unigenes matching to the same enzyme were considered as “and” relationship, and the unigenes matching to different enzymes of a reaction were considered to function independently, i.e., “or” relationship.

##### Confidence scoring system

According to the confidence rules in the paper of Thiele and Palsson (2010), the confidence score ranges from 0 to 4, where 4 represents direct evidence from biochemical data, 3 represents direct and indirect evidence from genetic data, 2 represents indirect evidence from physiological data and evidence for sequence data, 1 represents modeling needed data, and 0 represents no evaluated data. For Y-organ GSMN, the reactions in draft reconstruction were obtained based on the high-throughput sequencing data. Therefore, their confidence score is 2. The reactions added for the nutrient transport and added in the network evaluation step are needed for the synthesis of biomass and ecdysone, i.e., indirect evidence from physiological data, so their confidence score is also 2. The reactions added in the gap filling step are used for improving the network connectivity but has no evidence, so their confidence score is 1.

##### Determination of the biomass composition

The healthy crabs obtained from aquatic farm were cultured in a plastic incubator at 18–20°C for 5 days. During collection of the samples, crabs with average weight of 8–9 g were put on the ice plate for low-temperature anesthesia. The Y-organ of each crab was quickly cut off, placed in liquid nitrogen immediately, and then transferred to −80°C refrigerator for preservation. The biomass constituents in the samples were tested experimentally by the Beijing Institute of Nutritional Resources. The contents of 20 amino acids, 30 fatty acids, 10 trace elements, 2 carbohydrates, and 5 nucleotides were determined with the national standard testing method.

##### Adding biomass reaction

The biomass reactions were constructed according to the experimental results. The unit of substance content in the experimental results is g/100 g or mg/kg. The weight of water is firstly deduced from the total weight to get the weight of dry cell. Then the unit of each content was transformed to mmol/(g dry weight) to form the stoichiometry coefficient in the biomass reaction. The coefficients of ATP, ADP, and phosphate were set to be 29.8303 mmol/(g dry weight) for ATP maintenance referenced to *i*CHOv1 of *Cricetulus griseus* (Hefzi et al., 2016).

##### Adding transport and exchange reactions

The transport and exchange reactions were added according to the nutrient requirement of *E. sinensis*. Exchange reactions are the unbalanced network reactions that allow the accumulation of metabolites or provide the network with metabolites. Therefore, the transport and exchange reactions for the components composing the biomass were added, as well as H_2_O, H^+^, and O_2_, which are essential to most living beings.

##### Gap filling

In the draft reconstruction network, dead-end metabolites were unavoidable due to the incomplete understanding of gene annotation and metabolic system of an organism. The existence of dead-end metabolites may cause the breakpoints in some metabolic pathways, which leads to the failure of some metabolic functions. In order to reduce the dead-end metabolites and the breakpoints in the network, we try to fill the gaps by taking all the reactions in the KEGG database but not in the draft reconstruction network as the “metabolic environment.” The gaps were filled based on the graph theory and carried out in two scales: pathway scale (Hao et al., 2010) and global scale (Hao et al., 2012) successively. Taking the global scale gap filling as an example, the global network was firstly divided into different weakly connected components (WCCs). A WCC is defined as a network in which there is at least one path between any two nodes ignoring the direction of the edges, while different WCCs are not connected. The candidate reactions were thoroughly searched in the “metabolic environment” to connect different WCCs. If there are multiple candidate reactions to connect two WCCs, then the reaction with same or similar pathway with the reactions in the two WCCs was identified as gap reaction. For the pathway scale gap filling, each pathway in the network was divided into WCCs, and gaps inside the pathway were filled. With the addition of gap reactions, the breakpoints in the network were largely reduced.

#### Conversion of Reconstruction to Mathematical Model

To further refine and evaluate the reconstructed network, the reconstruction should be converted to a mathematical model. We used the COBRA toolbox (Heirendt et al., 2019), which can be used on Matlab platform to read and simulate the model. The biomass reaction was set to be the default objective function. The flux of reversible reactions was set to be (−1,000, 1,000) mmol gDW^–1^ h^–1^, and that for irreversible reactions is (0, 1,000) mmol gDW^–1^ h^–1^. The flux of transport reactions was set to be (−1,000, 1,000) mmol gDW^–1^ h^–1^. The exchange reactions for non-essential amino acid were set to be (0, 1,000) mmol gDW^–1^ h^–1^, which means that these amino acids do not need to be ingested from the environment. For the fatty acids that can be synthesized by the model, the related exchange reactions were set to be (0, 1,000) mmol gDW^–1^ h^–1^. The flux of other nutrient absorption was set to be (−5, 1,000) mmol gDW^–1^ h^–1^, except for trace elements, which is set to be (−1, 1,000) mmol gDW^–1^ h^–1^. The flux of H_2_O, H^+^, and O^2^ was set to be (−1,000, 1,000) mmol gDW^–1^ h^–1^. Finally, the refined network was converted into a Systems Biology Markup Language (SBML) formatted file, which can be read by the COBRA toolbox. The SBML file is valid by checking by MEMOTE (Lieven et al., 2020).

#### Network Evaluation

The network was evaluated by the non-essential amino acids, biomass, and ecdysone synthesis. The synthesis capability of these metabolites was calculated with flux balance analysis using COBRA toolbox.

There are 10 non-essential amino acids (alanine, asparagine, aspartic acid, cysteine, glutamine, glutamic acid, glycine, proline, serine, and tyrosine) in *E. sinensis*, which should be able to be synthesized by cells without being obtained from the feeds (Kanazawa and Teshima, 1981). The exchange reactions of these amino acids were set as the objective function. If a non-essential amino acid cannot be synthesized in the simulation (flux is 0), it means that the network is defective by missing one or more related reactions. The missing reaction(s) can be found by backtracking the related synthetic pathways of amino acids in databases or literatures. After the missed reactions are added, the accuracy and the synthetic ability of the model can be verified by recalculating the production of the amino acids. The revision was ended until all the 10 non-essential amino acids are correctly synthesized by the model. The ecdysone synthesis capability was evaluated in the same way.

For the biomass evaluation, biomass synthesis reaction was set to be the objective function. If the biomass cannot be synthesized (flux is 0), the synthesis of precursors used for biomass synthesis was checked. The transport and exchange reactions for the precursors were added firstly, and the flux of their exchange reactions was set to be (0, 1,000) mmol gDW^–1^ h^–1^, which means that the precursors are only allowed to be synthesized and not absorbed from the feeds. Then the exchange reaction of a precursor was set to be the objective function, and its maximum flux was calculated. When the precursor cannot be synthesized, the missing reactions were found by backtracking the pathways in the KEGG database and added to the model. The precursors were checked one by one until the biomass can be synthesized.

#### Data Assembly and Dissemination

Gap reactions obtained from the evaluation step were added to the model, and the feature of the network was counted and listed as the final reconstruction results.

### Topological Analysis

Currency metabolites such as ATP, ADP, NADH, NAD^+^, H_2_O, and H^+^, which are generally only transfer carriers of electron or some functional group and participate in a large number of reactions but do not directly participate in product synthesis (Ma and Zeng, 2003a), were removed from the reactions. With the removal of currency metabolites, the transformation of the major metabolites was shown to reveal more biological significance. Subsequently, the network was transformed into a reaction graph, that is, the network diagram with reaction as node and metabolite as edge. Then the topological features including density, average degree, average path length, diameter, cluster coefficient, and bow-tie structure were calculated with Pajek software (Batagelj and Mrvar, 2003). The bow-tie structure was analyzed based on the biggest WCC of the network.

### Network Decomposition

Network decomposition of the network is based on the combination of dendrogram and modularity (Ma et al., 2004; Hao et al., 2015). The biggest WCC of network, represented as a reaction graph, was layout as a classification tree with python package. A threshold of 50 for the maximal size of modules was set in the preliminary module partition by traversing the classification tree. Subsequently, a module with less than 10 reactions was combined with the largest module, which is connected with it. Finally, the optimal module partition was obtained by optimizing the modularity of the network. The degree of the nodes in the biggest WCC was calculated with Pajek software for the centrality analysis.

## Results and Discussion

### RNA Sequencing Results of Y-organ

**FIGURE 1:**
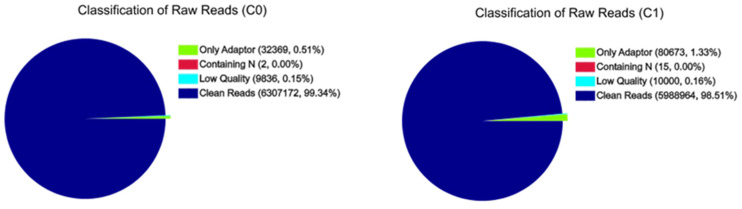
Classification of raw reads of samples C0 and C1.

**FIGURE 2:**
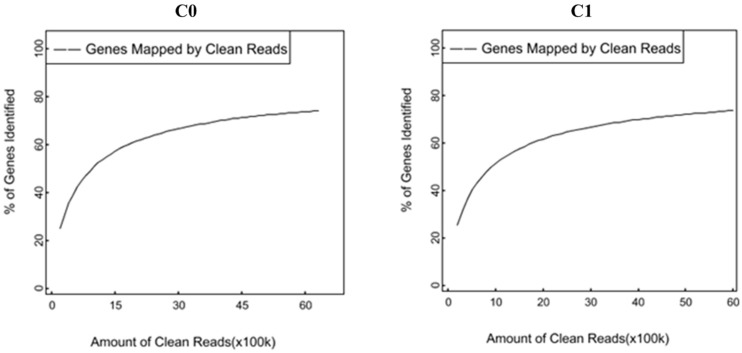
Sequence saturation analysis of samples C0 and C1.

**FIGURE 3:**
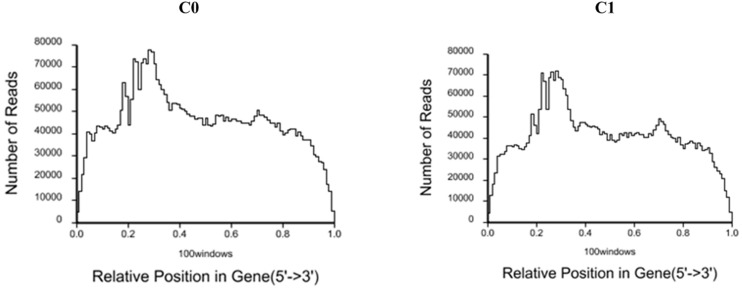
Distribution of reads in samples C0 and C1 on reference genes.

A total of 6,307,172 and 5,988,964 clean reads were obtained from C0 and C1 samples, accounting for 99.34 and 98.51% of the raw reads, respectively. The classification of raw reads is shown in Figure 1. These clean reads matched to 44,834 and 44,504 unigenes in the transcriptome data, respectively. Figure 2 shows the sequence saturation of samples C0 and C1. The number of detected genes tends to saturation in the two samples. Figure 3 shows the distribution of reads in samples C0 and C1 on reference genes. In both two samples, the reads in every position were evenly distributed, which indicate the good randomness of the sequencing.

### Reconstruction of the Y-organ Genome-Scale Metabolic Network

#### Draft Reconstruction

In the Y-organ RNA sequencing, there are a total of 44,834 unigenes obtained from sample C0, in which 12,581 unigenes have related KO information. Taking KO as a bridge, 1,885 unigenes in Y-organ were matched to the metabolic information in the KEGG database, and finally, 1,207 reactions and 958 metabolites were obtained.

#### Refinement of Reconstruction

In the determination of chiral metabolites, D-glucose in 13 reactions was replaced by α-D-glucose. Subsequently, reactions were searched in our previous reconstructed eyestalk and hepatopancreas GSMNs, and 1,192 reactions were found. The equation of these reactions in eyestalk and hepatopancreas GSMNs was used directly in the Y-organ model. The pathway and subsystem of all the reactions were obtained from the KEGG database, and all the 1,207 reactions were distributed in 98 pathways and 11 subsystems.

In the cellular composition testing, the content of a total of 59 nutrients was determined, including 20 amino acids, 30 fatty acids, 10 trace elements, 2 carbohydrates, and 5 nucleotides (Supplementary File S1). The total content of each kind of nutrient in 100-g cells is listed in Table 1. The unit of each nutrient was converted to mmol/(g dry weight), and the biomass equation was constructed (see Supplementary File S2, “Biomass” reaction).

**TABLE 1:** Content of each kind of nutrients in 100-g cells.

Nutrient	Protein	Lipid	Water	Carbon hydrate	Nucleotide	Trace element
Content	7.81 g	33.7 g	54.2 g	1.05 g	4.31 mg	0.74 g

The transport and exchange reactions for 20 amino acids, 30 fatty acids, 2 carbohydrates, and 10 trace elements were added. In addition, the transport and exchange reactions for H_2_O, H^+^, and O_2_ were also added, which is necessary for survival. A total of 130 reactions were added, and the number of reactions in the network increased to 1,337 after adding transport and exchange reactions.

Gap filling was processed in two scales, pathway scale and global scale. In the pathway scale, 105 gap reactions were added, and the number of reactions in the network increased from 1,337 to 1,442. In the global scale, 118 additional gap reactions were added. The number of WCCs of the network reduced from 151 to 87 after gap filling, which indicates that the gap filling step increased the network connectivity. After gap filling, the reconstructed metabolic network contains 1,560 reactions.

The refined network was transferred to .xml file, which can be read by COBRA toolbox and imported into the Matlab platform.

#### Network Evaluation

By investigating the synthesis capability of non-essential amino acids in *Eriocheir sinensis*, the quality of the reconstructed network was evaluated. The results showed that the simulated fluxes of two non-essential amino acids (cysteine and tyrosine) are 0, which means that these two amino acids cannot be synthesized by the network. By checking the cysteine metabolic pathway in the network and comparing it with that in the KEGG database, we found that the L-cysteine synthesis reaction R00897 (*O*-acetyl-l-serine + hydrogen sulfide <=> L-cysteine + acetate) was missed. Therefore, R00897 was added to the network. Furthermore, the transport and exchange reactions of sulfide were also added to ensure the positive flux of R00897. The synthesis of L-cysteine was simulated again, and the flux value increased from 0 to 5, indicating that cysteine can be successfully synthesized by the network. In the same way, R01728 [prephenate + NAD^+^ <=> 3-(4-hydroxyphenyl) pyruvate + CO_2_ + NADH + H^+^] were added to revise the tyrosine synthesis route in the network.

In the biomass simulation, the biomass was unable to be synthesized by the model at first. Therefore, the synthesis of all the precursors was checked. The problem occurs in the synthesis of some fatty acids because the fatty acid synthesis pathways in the network are incomplete. We checked the related KEGG pathways and added 29 metabolic reactions to complete the fatty acid synthesis route, including 26 reactions in “biosynthesis of unsaturated fatty acids” pathway and three reactions in “fatty acid biosynthesis” pathway. In addition, the transport and exchange reactions for 23 precursors of the fatty acids were added to check their synthesis capability, and the flux of exchange reactions was set to be (0, 1,000) mmol gDW^–1^ h^–1^. With the revision of fatty acid routes, the biomass was synthesized successfully, and the flux of biomass is 2.304 g h^–1^.

As ecdysone secretion is an important function of Y-organ, the quality of the model was further evaluated by the ecdysone synthesis. Since crab cannot synthesize cholesterol by itself but absorb it from the feeds (Hinsch, 1980), the transport and exchange reactions for cholesterol were firstly added, and the flux of exchange reactions was set to be (−5, 1,000) mmol gDW^–1^ h^–1^. In order to simulate the ecdysone synthesis, the transport and exchange reactions of ecdysone were also added with the flux of exchange reactions set to be (0, 1,000) mmol gDW^–1^ h^–1^. Then the exchange reaction of ecdysone was set to be the objective function, and the flux was calculated. The flux is not zero, which indicates that the ecdysone can be successfully synthesized by the network.

#### Data Assembly and Dissemination

**FIGURE 4:**
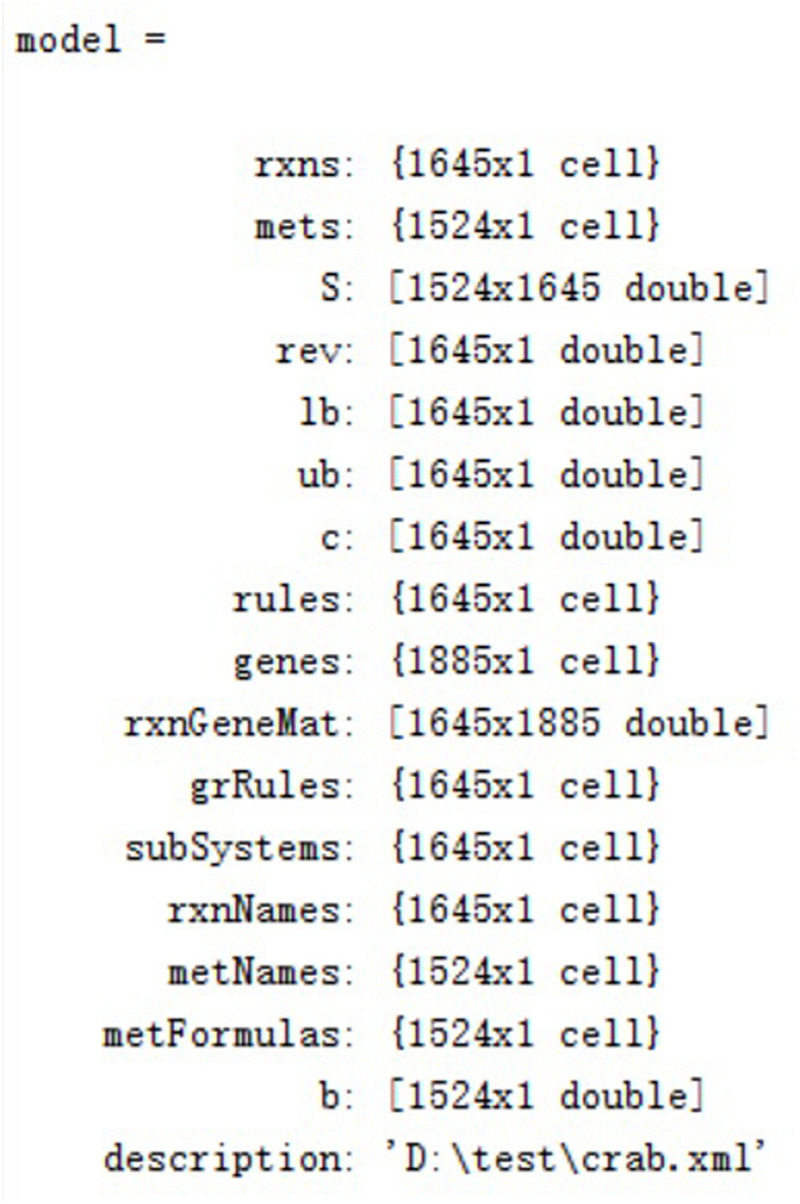
The components of model structure in Matlab.

The final reconstructed GSMN consists of 1,645 reactions, 902 enzymes, 1,524 metabolites, and 1,885 unigenes distributed in 100 pathways and 11 subsystems (Supplementary Files S2, S3). The characteristics of the GSMN are listed in Table 2. The components of model structure in Matlab are shown in Figure 4.

**TABLE 2:** The characteristics of Y-organ GSMN.

Items	Count
Unigenes	1,885
Reactions	1,645
Metabolic reactions	1,463
Transport reactions	91
Exchange reactions	91
Enzymes	902
Metabolites	1,524
Intracellular metabolites	1,421
Extracellular metabolites	103
Pathways	100
Subsystems	11

*GSMN, genome-scale metabolic network.*

### Topological and Functional Analyses of Reconstructed Genome-Scale Metabolic Network

#### Topology Analysis of Network Features

In all the 1,645 reactions in Y-organ GSMN, the currency metabolites and exchange reactions were firstly removed from the network, and some reactions with main reactions containing only currency metabolites were also removed. The finally reactions were 1,354, and the topological features of the network were calculated (Table 3). The GSMN of Y-organ was divided into 74 WCC, including 12 big WCCs with more than 10 reactions. There are 1,071 reactions in the largest WCC, accounting for 75.42% of the reactions in the network. The Y-organ network exhibits similar density and average degree with 15 tissues specific metabolic networks of human (with density in the range of 0.0016–0.0029 and average degree in the range of 3.43–4.51) (Hao et al., 2012). In addition, the biggest WCC clearly exhibits a typical bow-tie topology structure, which is proposed by Ma and Zeng (2003b) as an important feature of metabolic network. It is further proved by Zhao et al. (2006) that the bow-tie pattern is widely present in metabolic network with different chemical, spatial units, levels and sizes, and probably the result of evolution of metabolism in organisms. As an important hormone-secreting organ, Y-organ needs to exchange substances with other tissues and organs, import substrates, and export important precursors for hormone synthesis. The Y-organ network has higher proportion of giant strong component (GSC) than that of IN/OUT part, which indicates that Y-organ produces a small number of important precursors by consuming a large set of intermediates converted from a variety of inputs. The higher GSC proportion makes the Y-organ network have stronger resistance to perturbations. We further compared the clustering coefficient of the Y-organ network with 100 random networks with the same number of nodes and edges. We found that the Y-organ network has much higher clustering coefficient than random networks (0.371 vs. 0.00094∼0.00248), which indicates the small-world feature of the Y-organ network. This property is thought to enable metabolism to respond quickly to disturbances in enzyme or metabolite concentrations, so that cells can respond rapidly to environmental changes (Wagner and Fell, 2001).

**TABLE 3:** Basic topological features of Y-organ GSMN.

Parameter		Value
Nodes		1,420
	Arcs	2,528
	Edges	849
Density		0.0021
Average degree		4.7563
Average path length		10.1584
Diameter		29
Cluster coefficient		0.317
Biggest cluster	Nodes	1,071
	Arcs	2,248
	edges	793
Bow tie of the biggest cluster	GSC	492 (45.94%)
	S	201 (18.77%)
	P	225 (21.00%)
	IS	153 (14.29%)

*GSMN, genome-scale metabolic network.*

#### Function Analysis

##### Overview and analysis of main metabolic pathways

The 1,071 reactions in the largest WCC are distributed in 11 metabolic subsystems. Among these subsystems, amino acid metabolism contains the most reactions, followed by carbohydrate, lipid, and nucleic acid metabolisms (Table 4). These four metabolisms occupied 74.8% of the reactions in Y-organ GSMN. Tyrosine, arginine, proline, and cysteine metabolisms take up most of the reactions in the amino acid metabolism. The glycolysis, phosphoinositide metabolism, and tricarboxylic acid (TCA) cycle are the main pathways in the carbohydrate metabolism. Glycerol phosphate, sphingolipid, and arachidonic acid metabolisms occupied more than half of the total reactions in the lipid metabolism. Nucleic acid metabolism consists of purine and pyrimidine metabolism (Table 5). These metabolic processes all served as the main function of Y-organ to secrete various kinds of hormones.

**TABLE 4:** Reaction distribution in the largest WCC.

Subsystem	Number of reactions
Amino acid metabolism	276
Lipid metabolism	189
Carbohydrate metabolism	183
Nucleotide metabolism	153
Metabolism of cofactors and vitamins	74
Biosynthesis of secondary metabolites	57
Transport	56
Xenobiotics biodegradation and metabolism	37
Translation	21
Energy metabolism	20
Glycan biosynthesis and metabolism	5
Total	1,071

*WCC, weakly connected component.*

**TABLE 5:** The main pathways in different metabolic subsystems.

Subsystem	Main pathways	Reactions	Proportion
Amino acid	Tyrosine metabolism	45	16.3%
metabolism	Arginine and proline metabolism	35	12.7%
	Valine, leucine, and isoleucine degradation	28	10.1%
Carbohydrate	Glycolysis	27	14.8%
metabolism	Inositol phosphate metabolism	26	14.2%
	TCA cycle	16	8.7%
Lipid metabolism	Glycerol phosphate metabolism	37	19.6%
	Biosynthesis of unsaturated fatty acids	25	13.2%
	Sphingolipid metabolism	23	12.2%
Nucleic acid metabolism	Purine metabolism	85	55.6%
	Pyrimidine metabolism	68	44.4%

*TCA, tricarboxylic acid.*

Tyrosine was a precursor of catecholamines (such as dopamine, adrenaline, and noradrenaline), which enables the cell to make emergency responses to the external environment. Therefore, tyrosine played an essential role in the metabolism of animals (Xiong, 2013). In the reconstructed GSMN, we found the complete synthetic route from tyrosine to adrenaline (L-tyrosine → dopamine → noradrenaline → L-adrenaline).

Both glycolysis and TCA cycle were ubiquitous metabolic pathways in cells. In the reconstructed network, we found the hexokinase (K00844, R01600), 6-phosphofructokinase-1 (K00850, R04779), and pyruvate kinase (K00873, R00200) in the glycolysis pathway; and citrate synthase (K01647, R00351), isocitrate dehydrogenase (K00030, R00709), and α-ketoglutarate dehydrogenase (K00164, R00621; K00658, R02570; K00382, R03815) in the TCA circle pathway. Reactions catalyzed by these enzymes constitute the core part of the carbohydrate metabolism. Besides, in the carbohydrate metabolism subsystem, inositol phosphate metabolism accounts for 14.4% of the carbohydrate metabolism. This pathway is about a series of reactions to generate two second messengers, inositol triphosphate (IP_3_), and diacylglycerol (DAG), from phospholipase C (K01114). The main function of IP_3_ is to regulate the release of Ca^2+^, whereas DAG regulates the activity of protein kinase K, which played an important role in cell proliferation, differentiation, contraction, and metabolism (Vines, 2012).

The important function of Y-organ is to secrete ecdysone. In the KEGG database, this process belongs to the insect hormone biosynthesis pathway in the metabolism of terpenoids and polyketides. Since crab cannot synthesize cholesterol by itself but absorb it from feeds (Hinsch, 1980), the ecdysone synthesis pathway in crabs begins with cholesterol and generates 7-dehydrocholesterol (7DC) catalyzed by neverland (*nvd*) gene (Yoshiyama et al., 2006). Then 7DC turns into 5β-diketol (3D2, 22, 25, dE) under the function of *spook* (related to CYP307A1), *spookie*r (related to CYP307A2), and *spookiest* (related to CYP307B1) genes (Thummel and Chory, 2002; Zhou and Zhu, 2016). The *5*β*-diketol* is then used to synthesize ecdysone catalyzed by CYP306A1, CYP302A1, and CYP3115A1, which are coded by *phantom*, *gisembodied*, and *shadow* genes (Böcking et al., 1993). Finally, the activated 20-hydroxyecdysone is produced (Petryk et al., 2003; Rewitz et al., 2006) from ecdysone catalyzed by CYP314A1. *neverland* (*nvd*), *spook* (*spo*), *spookier* (*spok*), *spookiest* (*spokest*), *phantom* (*phm*), *disembodied* (*dib*), *shadow* (*sad*), and *shade* were a series of genes named by Nüsslein and his colleagues for investigating the mutation trait of *Drosophila*. They were called “Halloween Family Gene” and play significant role in the synthesis of ecdysone (Nusslein-Volhard, 1980). In crustaceans, Xie et al. (2016) successfully cloned the *disembodied* (*dib*), *spook* (*spo*), and *shadow* (*sad*) genes from *Portunus trituberculatus* (Liu et al., 2015), and Chang and O’Connor (1977) found that the expression level of these genes in Y-organ was significantly higher than that of other tissues, which was consistent with the conclusion that the Y-organ is the primary secretory organ of crustacean synthetic ecdysone.

**FIGURE 5:**
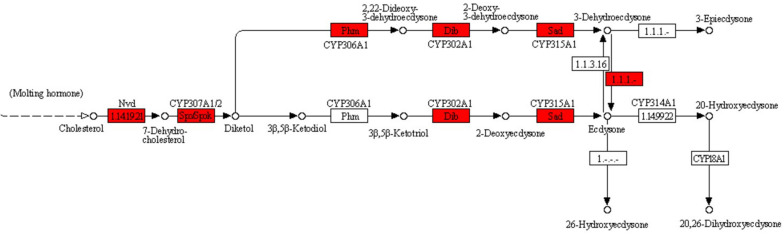
Pathway for synthesizing ecdysone from cholesterol. Red-labeled enzymes (encoded by *nvd*, *spo*, *spok*, *dib*, and *sad*) are found in Y-organ genome-scale metabolic network (GSMN), while the *phm*-related reaction R08136 was added to the network during the gap filling process.

In the GSMN of *E. sinensis* Y-organ, we found six genes (enzymes) (encoded by *nvd*, *spo*, *spok*, *dib*, *sad*, and *phm*) and their related reactions in the ecdysis pathway (Figure 5). The related reactions of *nvd*, *spo*, *spok*, *dib*, and *sad* were collected in the preliminary reconstruction of the network, while the *phm*-related reaction R08136 was added to the network during the gap filling process. However, we did not find the related reactions about CYP314A1. This enzyme maybe lost in the high-throughput sequencing process and needs to be further studied.

##### Module analysis

**FIGURE 6:**
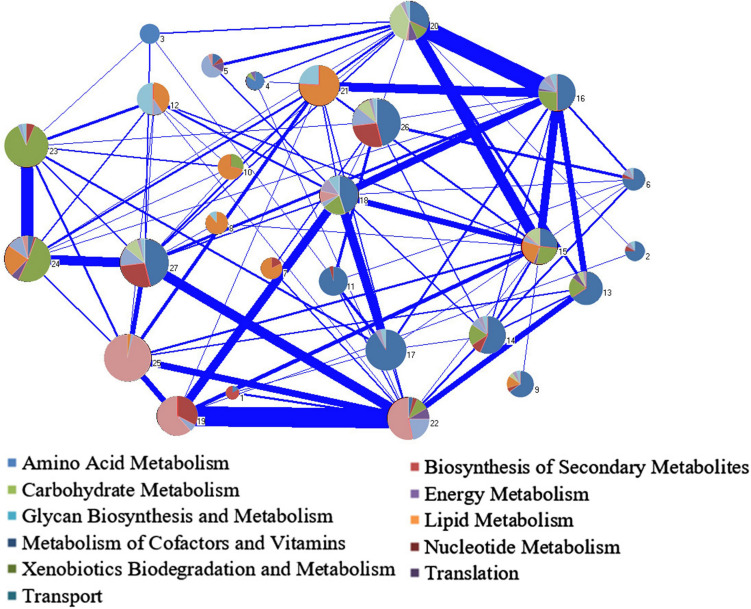
Module decomposition of the biggest weakly connected component (WCC) in Y-organ genome-scale metabolic network (GSMN). Each pie chart represents a single module and contains several different subsystems. The area of a color shows the percentage of the subsystem it represents in the pie chart. The width of the line between the modules represents the strength of connection.

In the network decomposition, the largest WCC was decomposed into 27 modules, of which seven modules (modules 1, 3, 7, 8, 10, 11, and 21) contained only one metabolic subsystem, and most of the modules (15/27) contained less than three metabolic subsystems (Supplementary File S3). It indicates that the relative independence of each module and each module represents a certain biological function. There are 83 reactions in module 27, including 7 subsystems, which contains the most subsystems. It is speculated that the completion of the specific physiological function of this module required cooperation of multimetabolic pathways. Many reactions in the carbohydrate pathways were included in module 27. Since the intermediate products of central carbon metabolism are important precursors of many metabolic pathways, it is reasonable that module 27 contains multiple subsystem metabolic pathways. The connections between modules indicate the material exchanges and cooperation between different modules to accomplish their functions (Figure 6). The connections between modules with more subsystems were generally stronger.

##### Centrality analysis

The reaction with larger centrality indicates that it is more closely linked to other reactions and has greater importance in the metabolic system (Wagner and Fell, 2000). Degree is one of the features to reflect the centrality of the nodes. In the biggest WCC, R00899 (Cys-Gly + H_2_O <=> L-cysteine + glycine) is the reaction with the largest degree, which belongs to amino acid metabolism subsystem in module 20. Cys-Gly is the hydrolyzed product of glutathione (GSH), which is a tripeptide substance composed of glutamate, cysteine, and glycine. GSH has antioxidant effects and plays an important role in scavenging free radicals (Kortenkamp and O’Brien, 1994), biological detoxification enhancing antioxidant enzyme activity, regulating immune system, and enhancing anti-stress ability in aquatic animals (Xu et al., 2012). L-Cysteine is an important metabolic intermediate of many amino acids. In addition, it can be degraded into another import intermediate product in the carbohydrate metabolism, pyruvate, which mediates the mutual conversion of carbohydrate, fats, and amino acids in cells through acetyl-CoA and TCA cycles (Covi et al., 2009; Yizhou Qi et al., 2010). Therefore, R00899 locates in the center of the network and plays an essential role in the whole metabolisms.

### Module Analysis of Differentially Expressed Unigenes

We obtained a total of 1,918 DEUs between groups C0 (control group) and C1 (eyestalk ablation group), according to the selection criterions described in the *Methods* section (Supplementary File S5). Among these DEUs, 474 unigenes were found in the reconstructed GSMN, and 405 were included in the largest WCC (Table 6). The reactions catalyzed by the enzymes encoded by the DEUs were considered as differential reactions.

**TABLE 6:** Results of differential genes expression experiment.

	Number of unigenes		
DEUs	**1,918**	Up	898
		Down	530
DEUs in GSMN	**474**	Up	191
		Down	283
DEUs in the biggest WCC	**405**	Up	144
		Down	261

*DEU, differentially expressed unigene; GSMN, genome-scale metabolic network; WCC, weakly connected component.*

We extracted the modules with the most differential reactions in each subsystem, and their related metabolic pathways were analyzed. The differential reactions of the amino acid metabolism, carbohydrate metabolism, and lipid metabolism were mainly in modules 14 and 27, including valine, leucine, and isoleucine degradation; inositol phosphate metabolism; and glycerophospholipid metabolism. Module 22 contains most of the differential reactions in nucleic acid metabolism and all the differential reactions in purine metabolism. The differential reactions of cofactor and vitamin metabolism, energy metabolism, and translate subsystem were mainly in modules 3 (porphyrin and chlorophyll metabolism), 22 (sulfur metabolism), and 18 (aminoacyl tRNA synthesis). These modules and pathways show the most responsive metabolic processes in Y-organ influenced by the eyestalk ablation.

There are also DEUs in the ecdysis pathway, including R11007 (cholesterol <=> 7-dehydrocholesterol, Unigene39444_A0A, down-regulation); R08132 (7-dehydrocholesterol <=> diketol, Unigene19254_A0A, up-regulation), R08133, R08134 (diketol <=> 3beta, 5beta-Ketotriol <=> 2-deoxyecdysone, Unigene1867_A0A, up-regulation), and R08135 (2-deoxyecdysone <=> ecdysone, Unigene45919_A0A, up-regulation). The expression of genes corresponding to ecdysone production from 7-dehydrocholesterol was all up-regulated, indicating that eyestalk ablation accelerated the synthesis of ecdysone and the process of ecdysis. This phenomenon was reported by Zeleny firstly (Zeleny, 1905), and many researches got similar results in crustaceans, such as *Penaeus chinensis* (Chu and Chow, 1992), *Macrobrachium nipponense* (Zhang et al., 1999), *Procambarus clarkia* (Yin, 2007), *Sinopotamon henanense* (Pei et al., 2014), and *E. sinensis* (Cui et al., 2004). After eyestalk ablation, the secretion of ecdysone increases in a short period, which accelerates the ecdysis, finally, promoting the growth of crab. In addition, the expression level of *spook* gene and *disembodied* gene had a good cooperative relationship, which are all up-regulated in C1 group, suggesting that *spook* gene, as an early synthetase of ecdysone, has a close relationship with the downstream synthetase and ecdysone synthesis, which is consistent with the work of Wang Chun-Jian et al. (2013).

### Comparison of Y-organ With Eyestalk and Hepatopancreas Genome-Scale Metabolic Networks

#### Comparison of Model Features

We compared the Y-organ GSMN with the eyestalk and hepatopancreas GSMNs reconstructed in our previous study (Wang B. et al., 2016; Hao et al., 2018). The numbers of unigenes in Y-organ eyestalk and hepatopancreas transcriptome are 44,834, 48,835, and 41,147. The numbers of unigenes and reactions in Y-organ are similar with those in hepatopancreas, but much more than those in eyestalk. Most of the pathways in the three networks are common, but each organ has its specific pathway(s). The biosynthesis pathway of tropane, piperidine, ecdysone, and pyridine alkaloids is unique in the Y-organ network. The D-glutamine and glutamic acid metabolisms exist only in the eyestalk network. The basic features of the three networks are shown in Table 7.

**TABLE 7:** Basic features of the eyestalk and Y-organ GSMN.

Items	Y-organ	Eyestalk	Hepatopancreas
Genes	1,885	1,381	1,882
Reactions	1,645	1,304	1,471
Metabolic reactions	1,463	1,250	1,417
Transport reactions	91	27	27
Exchange reactions	91	27	27
Metabolites	1,524	1,243	1,400
Intracellular metabolites	1,421	1,214	1,371
Extracellular metabolites	103	29	29
Pathways	100	100	101
Subsystems	11	11	11

*GSMN, genome-scale metabolic network.*

The topological features of the three GSMNs were further compared (Table 8). The scale of Y-organ and hepatopancreas GSMNs is similar but larger than that of eyestalk after removing the currency metabolites and exchange reactions. The largest WCC of Y-organ contains more reactions than that of eyestalk and hepatopancreas (1,071 vs. 797 and 991). The diameter of eyestalk network is smaller than that of Y-organ and hepatopancreas (21 vs. 29 and 32). The three networks have a typical bow-tie structure. In the GSC of the bow-tie structure, the Y-organ, eyestalk, and hepatopancreas networks include 492, 394, and 476 reactions, taking 45.94, 49.4, and 48% of the total reactions, respectively. The density, average degree, and clustering coefficients of the three organs are similar. It is reasonable considering that they come from the same species.

**TABLE 8:** Topological features of Y-organ, eyestalk, and hepatopancreas GSMNs.

Parameter		Y-organ	Eyestalk	Hepatopancreas
Nodes		1,420	1,128	1,342
Arcs		2,528	1,970	2,314
Edges		849	769	1,012
Density		0.00219	0.002757	0.002409
Average degree		4.7563	4.856383	4.956781
Average path Length		10.158	8.9510	9.8294
Diameter		29	21	32
Cluster coefficient		0.317	0.33922	0.33922
Biggest WCC	Nodes	1,071	797	991
	Arcs	2,248	1,702	2,030
	Edges	793	717	937
Bow-tie of biggest WCC	GSC	492	394	476
	S	201	75	178
	P	225	253	156
	IS	153	75	181

*GSMN, genome-scale metabolic network; WCC, weakly connected component.*

#### The Common Metabolisms of Y-organ, Eyestalk, and Hepatopancreas Genome-Scale Metabolic Networks

**FIGURE 7:**
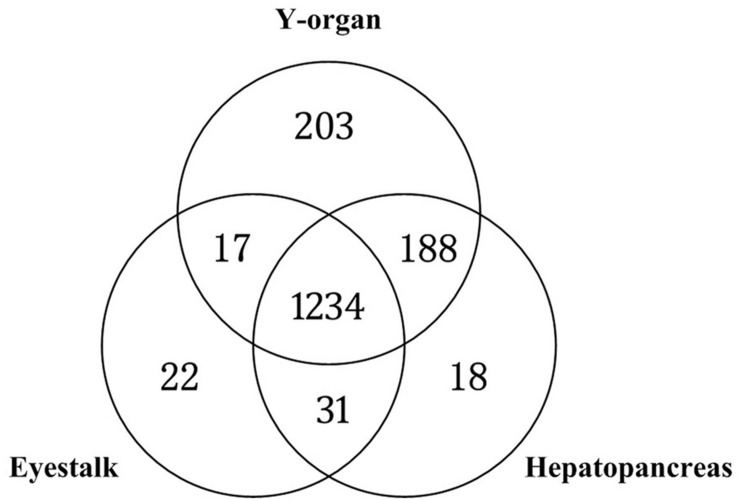
Comparison of Y-organ, eyestalk, and hepatopancreas genome-scale metabolic networks (GSMNs).

The metabolic reactions of Y-organ, eyestalk, and hepatopancreas GSMNs were compared, and 1,182 common reactions were found (Figure 7). There are 203, 22, and 18 specific reactions in the Y-organ, eyestalk, and hepatopancreas networks, respectively.

The common metabolic reactions in the three networks are distributed in 11 subsystems. The subsystems that contain the most reactions are amino acid, lipid, carbohydrate, and nucleic acid metabolisms. This result is consistent with the subsystems that have the most reactions in the largest WCC of Y-organ GSMN. The pathways contain more than 30 reactions that belong to nucleic acid metabolism, amino acid metabolism, lipid metabolism, xenobiotics biodegradation, and metabolism subsystems. These common reactions in the three networks are mainly associated with the basic metabolic processes in cells.

#### Unique Metabolisms in Each Genome-Scale Metabolic Network

##### The unique metabolisms in *Eriocheir sinensis* Y-organ genome-scale metabolic network

There are 36 specific metabolic reactions in the Y-organ network after excluding exchange and transport reactions, which are distributed in 16 pathways. The pathway that contains the most unique reactions is the glycerol phospholipid metabolism, which contains seven unique reactions. These reactions include the lecithin synthesis reaction from diglyceride and phosphatidylserine synthesis reaction from lecithin catalyzed by phosphatidylserine synthase 1.

In the tryptophan metabolism pathway in the Y-organ network, the important neurotransmitter, serotonin, was found to be synthesized from 5-hydroxytryptophan catalyzed by aromatic L-amino acid decarboxylase DDC (EC: 4.1.1.28) (R02701). In phenylalanine metabolism pathway, the main specific reactions of Y-organ are the decomposition of L-phenylalanine to phenylethylamine (R00699) and the formation of 2-hydroxyphenylacetic acid from phenylpyruvate (R01372). In the alanine, aspartic acid, and glutamate metabolism pathways, the specific reactions are mainly concentrated in the pathway related to aspartic acid synthesis and metabolism (R00485, R00578).

The related reactions of lecithin synthesis and metabolism (R05794, R07377) were found specific in Y-organ. Lecithin (phosphatidylcholine) is the main lipid of cell membrane (Kenari et al., 2011), which plays a role in lipoproteins, synthesis, and secretion (Coutteau et al., 2000). Wang J. T. et al. (2016) found that *P. trituberculatus* can significantly promote the growth and molting of animals by supplementing 20 g/kg of lecithin every day for 50 consecutive days.

5-Hydroxytryptamine (5-HT) is widely distributed in the central and peripheral nerve tissues of crustaceans. As a neurotransmitter, neuromodulator, or hormone, it plays an important role in regulating the behavior, reproduction, molting, digestion, absorption, and metabolism of animals (Fingerman et al., 1994; Sloley and Juorio, 1995). Sun et al. (2006) studied the effect of 5-HT on the excitability and secretory activity of neuroendocrine cells in the eyestalk of crab by using the whole cell patch-clamp technique. It was found that 5-HT can regulate the blood glucose level and molting activity of hemolymph of crab through the eyestalk neuroendocrine system. The reaction of synthesizing 5-HT from 5-hydroxytryptophan catalyzed by aromatic L-amino acid decarboxylase DDC (EC: 4.1.1.28) was found in Y-organ as a specific reaction, which indicates that the Y-organ of *E. sinensis* has the ability to synthesize 5-HT.

The Y-organ GSMN also contains the metabolic pathway of cytochrome P450 (R00711, R00712). Cytochrome P450 enzyme system (CYP) is a kind of metabolic enzyme system widely existing in various organisms (Feyereisen, 1999), which is involved in the transformation and metabolism of ecdysone, fatty acid, and juvenile hormone (Simpson, 1997) and plays an important role in the synthesis and degradation of 20 hydroxyecdysterone (Gilbert and Rewitz, 2009). Xia et al. (2012) compared the relationship between CYP4V18 gene expression and 20-hydroxyecdysterone titer in the blood of *Litopenaeus vannamei*. The results showed that CYP4V18 down-regulation could lead to the increase of ecdysterone concentration, which is helpful to the ecdysis process in crustaceans.

In addition, the important reaction (R08132) synthesizing diketol from 7-dehydrocholesterol (7DC) catalyzed by *spook* (CYP307A1) and *spookier* (CYP307A2) is also found as a specific reaction in Y-organ.

##### The unique metabolisms in *Eriocheir sinensis* eyestalk genome-scale metabolic network

There are 22 specific reactions in eyestalk GSMN distributed in 11 different pathways. The glycine, serine, and threonine metabolisms contain more than five unique reactions, which are all reactions producing betaine aldehyde from choline (choline < = > betaine aldehyde). These five reactions are catalyzed by four different enzymes including choline monooxygenase (EC:1.14.15.7), choline oxidase (EC:1.1.3.17), choline dehydrogenase (EC:1.1.99.1), and alcohol dehydrogenase (EC:1.1.1.1). The produced betaine aldehyde can be further oxidized to betaine, which is an effective osmoregulation substance, helping to reduce the osmolality caused by the change of salt concentration and improve the survival rate of fish in the process of migration from fresh water to sea water. Virtanen et al. (1994) found that by adding betaine to rainbow trout, the content of sodium and magnesium ions in plasma will be changed, so as to improve the adaptability to the environment. At the same time, betaine, as a methyl provider, can be used as bait attractant to improve feed intake. Peñaflorida and Virtanen (1996) found that by adding the mixture of amino acid and betaine, the growth rate of juvenile shrimp can be increased. Choline is a synthetic precursor of many phospholipids, such as lecithin, lysophosphatidylcholine, and sphingomyelin. These phospholipids are important components of cell membrane. In addition, they also exist in the cytoplasm. As the components of lipoproteins, choline is responsible for the transport of lipids between tissues and organs (Simon, 1999).

In the amino sugar and nucleic metabolism pathways, four unique metabolic reactions (R01805, R02087, R02705, and R04435) were found. These reactions generate sialic acid from *N*-acetyl-D-glucosamine 6-phosphoric acid (GlcNAc-6P). Sialic acid is a general term of derivatives of 9-carbomonosaccharide family (Schauer, 2004), which usually exists at the end of glycoconjugates on the cell surface and is an important part of lectin ligand recognition (Simon, 1999). Among the crustaceans, sialic acid lectin has been extracted from *Homarus americanus* (Hall and Rowlands, 1974), *Macrobrachium rosenbergii* (Vasta et al., 1983), *Cancer antennarius* (Ravindranath et al., 1985), *Scylla serrate* (Mercy and Ravindranath, 1993), and *Penaeus monodon* (Ratanapo, 1990). Denis et al. (2015) extracted sialic acid-specific lectin from blood lymphocytes of *Paratelphusa jacquemontii* using bovine submandibular gland mucus as an affinity chromatography ligand. In the GSMN of *E. sinensis* eyestalk, we found the synthetic reactions of sialic acid from *N*-acetyl-D-glucosamine-6-phosphate, which suggests that there are sialic acid synthesis pathway and corresponding functions in *E. sinensis*. This result provides a reference for the further research on the neuroendocrine regulation and development of the eyestalk.

##### The unique metabolisms in *Eriocheir sinensis* hepatopancreas genome-scale metabolic network

There are 18 metabolic reactions specific to hepatopancreas, which are distributed in 12 pathways. Among them, there are four reactions in the GSH metabolic pathway, all of which are related to *Trypanosoma* cysteine synthesis and metabolism. *Trypanosoma* cysteine is a dimer of two GSH molecules linked by spermidine. It was first found in *Leishmania*, *Trypanosoma*, and other Protozoa (Fairlamb and Cerami, 1992). GSH is a tripeptide composed of glutamic acid, cysteine, and glycine, which mainly exists in the hepatopancreas, muscle, and blood. It has an antioxidant effect and can regenerate other antioxidants, such as VC, VE, β-carotene, and Se cysteine. GSH, as a non-enzymatic antioxidant, plays a role in scavenging free radicals *in vivo*. In addition, it also has the function of biological detoxification and is the biological reductant of Cr^6+^ in cells (Kortenkamp and O’Brien, 1994). GSH can be added to the feed of fish and crustacean, which has a positive effect on the endocrine of aquatic animals. Adding GSH to the feed of tilapia and *L. vannamei* can improve the digestibility of the feed and the growth rate and survival rate. In addition, GSH plays an important role in improving the activity of antioxidant enzymes, regulating the immune system, and enhancing the anti-stress ability of aquatic animals (Xu et al., 2012).

## Conclusion

The GSMNs have been established for many organisms and applied to solve many metabolic issues. In this work, a GSMN of Y-organ in *Eriocheir sinensis* was reconstructed and validated according to the standard protocol. Module analysis and differential unigene analysis of the network revealed the main function of Y-organ. The comparison of the GSMNs of Y-organ, eyestalk, and hepatopancreas brings insight into the specific metabolic pathways in each organ. Due to the lack of experimental data, the validation of the *E. sinensis* GSMN cannot be as sufficient as many model organisms, but the establishment of the GSMN still provides an important platform for the study of the metabolic process of *E. sinensis* and even aquatic crustaceans, which is an important step in the process from *in vivo* to *in silico* experiment in aquatic crustacean research. With the help of the GSMN, the *in vivo* experiment will develop more rapidly, which will further promote the development of the aquatic crustaceans GSMNs and improve the quality of the networks.

## Data Availability Statement

The datasets generated for this study can be found in the SRA database: SRX8639987, SRX8639988; GEO database: GSE153552.

## Ethics Statement

The animal study was reviewed and approved by the Tianjin Normal University.

## Author Contributions

BW drafted the manuscript and performed the network reconstruction and analysis. JY and CG prepared the database and participated in the data analysis. JL performed the biomass composition analysis. TH and JS contributed to the guideline and revision of the manuscript. All authors read and approved the final manuscript.

## Conflict of Interest

The authors declare that the research was conducted in the absence of any commercial or financial relationships that could be construed as a potential conflict of interest.

## Footnotes

1 https://www.ncbi.nlm.nih.gov/sra/SRX8639987[accn]

2 https://www.ncbi.nlm.nih.gov/sra/SRX8639988[accn]

3 https://www.ncbi.nlm.nih.gov/geo/query/acc.cgi?acc=GSE153552

## Supplementary Material

Supplementary File 1 Biomass compositions

Supplementary File 2 GSMN of Y-organ. “reactions” worksheet includes information of reactions, and “metabolites” worksheet includes information of metabolites in the network.

Supplementary File 3 SBML formatted file of Y-organ GSMN.

Supplementary File 4 Results of network decomposition.

Supplementary File 5 The DEUs between group C0 and C1.
